# Preparation, molecular docking, and biological evaluation of radioiodinated cefaclor for inflammation detection

**DOI:** 10.1186/s40360-026-01117-z

**Published:** 2026-03-27

**Authors:** H. Hussien, Marwa S. El Refaye, H. Aglan, Safaa B. Challan, S. I. Khater

**Affiliations:** 1 Labeled Compounds Department, Hot Laboratories and Waste Management Center, Egyptian Atomic, Energy Authority, P.O. Box 13759, Cairo, Egypt; 2 Cyclotron Facility, Nuclear Research Center, Egyptian Atomic Energy Authority, P.O. Box 13759, Cairo, Egypt; 3 Radioactive Isotopes and Generators Department, Hot Laboratories and Waste Management Center, Egyptian Atomic Energy Authority, P.O. Box 13759, Cairo, Egypt

**Keywords:** Cefaclor, Iodine-131 [^131^I], Iodogen, Molecular modeling, Molecular docking, Biological evaluation, Septic and aseptic inflammation

## Abstract

**Background:**

Inflammation serves as a natural defense mechanism; however, its persistence can lead to chronic diseases with serious clinical consequences. Early detection of inflammation is therefore critical to slowing disease progression and improving therapeutic outcomes.

**Methods:**

In this study, cefaclor (Cefa) was successfully radiolabeled with iodine-131 via electrophilic substitution to facilitate the detection of infected and inflamed muscles in mouse models. The labeling reaction was carried out with 100 µg of Cefa and 100 µg of iodogen, using glass frits as the oxidizing system at pH 7 and 60 °C, with 10 µL of Na^131^I for 20 min. The resulting [^131^I]Cefa was purified by high-performance liquid chromatography (HPLC). Molecular modeling was performed in the Molecular Operating Environment (MOE) to evaluate the compound’s structure and binding affinity.

**Results:**

The labeling process was optimized to achieve a radiolabeling efficiency of 90 ± 0.56%, and stability of about 89 ± 0.5% at 4 h. Docking simulations confirmed strong binding of [^131^I]Cefa to bacterial DNA gyrase B, supporting its potential as a targeted imaging agent. Biological evaluation in mouse models demonstrated notable tracer accumulation in both septic and sterile inflammatory sites. Uptake values reached 28 ± 1.5%ID/organ in infected muscles and 16 ± 1.5%ID/organ in sterile inflammation at 120 minutes post-injection. The target-to-non-target (T/NT) ratios were 5.28 for infected muscles and 2.1 for sterile inflammation, indicating effective differentiation between bacterial (septic) and non-bacterial (aseptic) inflammatory foci.

**Conclusion:**

The radiolabeled [^131^I]Cefa compound exhibits promising diagnostic capabilities for distinguishing bacterial infections from sterile inflammation. Its high radiolabeling efficiency, strong molecular binding, and selective in vivo uptake support its potential utility as a non-invasive imaging agent for early detection and characterization of inflammatory conditions.

## Highlights

Objective: To develop a radiolabeled cefaclor compound ([^1^^3^^1^ I] Cefa) for early imaging and differentiation between bacterial infections and sterile inflammation.

Radiolabeling Method: Cefaclor was labeled with iodine-131 via electrophilic substitution, achieving a high radiochemical yield of 90 ± 0.56% using iodogen and glass frits as the oxidizing system.

Structural Analysis: Molecular modeling and docking simulations confirmed strong binding of [^1^^3^^1^ I] Cefa to bacterial DNA gyrase B, supporting its role as a targeted imaging agent.

In Vivo Findings: In mouse models, the compound showed significant uptake in infected (28± 1.5%) and sterile inflamed tissues (16 ± 1.5%) at 120 minutes post-injection.

Diagnostic Value: The target-to-non-target ratios (28 for infection, 2.1 for sterile inflammation) demonstrate the compound’s potential to distinguish between infectious and non-infectious inflammation.

## Introduction

Inflammation is a fundamental biological response to harmful stimuli, including pathogens, toxins, or cellular damage, functioning as a critical defense mechanism to restore tissue homeostasis. Characterized by the activation of immune cells, the release of cytokines, and increased vascular permeability, acute inflammation is typically self-limiting and protective. However, when deregulated, inflammation can transition into a chronic state, perpetuating tissue damage and contributing to the pathogenesis of numerous diseases, such as rheumatoid arthritis, atherosclerosis, diabetes, and cancer [1]. Infectious diseases caused by Gram-negative bacteria, such as *Escherichia coli (E. coli)*, represent a significant global health burden, ranging from urinary tract infections to life-threatening sepsis [2]. The emergence of multidrug-resistant *E. coli* strains has further complicated treatment strategies, necessitating improved diagnostic tools and enhanced therapeutic monitoring. Inflammatory responses triggered by such infections often exacerbate tissue damage and systemic complications, underscoring the need for advanced imaging modalities capable of capturing real-time molecular events [3].

The incorporation of radionuclides, such as ^99m^Tc, ^125^I, and ^131^I, represents a cornerstone in the synthesis of molecular imaging agents. Among these, iodine-131 [^131^I] stands out due to its bifunctional emission profile encompassing both beta and gamma radiation, which facilitates its application in both diagnostic imaging and therapeutic interventions [4–6]. This isotope confers multiple benefits, including straightforward manipulation, economic viability, and minimal environmental impact, thereby positioning it as a preferred radiolabel for innovative pharmacophores. The radiolabeling procedure generally proceeds through electrophilic aromatic substitution, necessitating the formation of a robust conjugate exhibiting elevated radiochemical purity to ensure suitability for subsequent biodistribution analyses and imaging protocols [7]. A diverse array of pharmaceutical agents has been utilized in previous studies for inflammation imaging, such as^131^I-ornidazole, ^131^I-oseltamivir phosphate,^125^I-amoxicillin^99m^Tc-graphene oxide,^99m^Tc-teicoplanin,^99m^Tc-ceftazidime,^99m^Tc-vancomycin, ^99m^Tc-cefoperazone, ^99m^Tc-kanamycin, ^99m^Tc-alafosfalin, and ^99m^Tc-ciprofloxacin [8–18].

**Fig. 1:**
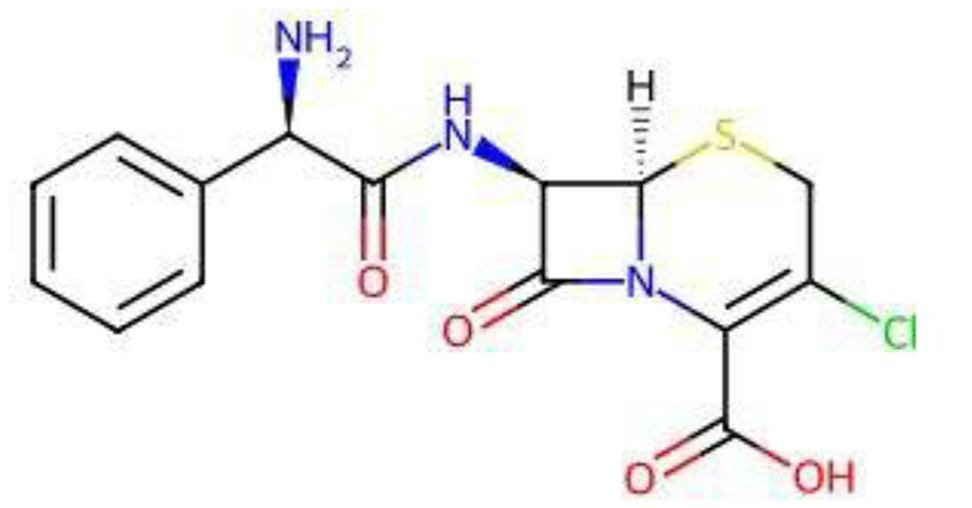
Structure of cefaclor

The chemical designation of cefaclor (Cefa) is (*6 R,7 R*)-7-{[(*2 R*)-2-Amino-2-phenylacetyl]amino}-3-chloro-8-oxo-5-thia-1-azabicyclo[4.2.0]oct-2-ene-2-carboxylic acid, as depicted in Fig. 1 [19]. It represents a second-generation semisynthetic cephalosporin antibiotic that is utilized for the treatment of various bacterial infections and demonstrates broad-spectrum efficacy against both Gram-positive and Gram-negative pathogens, including *E. coli* [20]. Its molecular architecture comprises a β-lactam ring fused to a dihydrothiazine ring, with substitutions at R1 and R2 that enhance its antimicrobial potency. The β-lactam moiety facilitates irreversible inhibition of bacterial cell wall synthesis by targeting penicillin-binding proteins (PBPs), resulting in osmotic imbalance and bacterial lysis [21].

This study investigates the radiolabeling of cefaclor with iodine-131 via electrophilic aromatic substitution, optimizing parameters that influence the radiolabeling efficiency. Theoretical modeling using the Spartan program is employed to predict the most stable structure of [^131^I]Cefa. Radiochemical purity is evaluated using high-performance liquid chromatography (HPLC), and biodistribution studies are conducted in normal, infected (E. coli- induced), and inflamed mice to assess the tracer'sdiagnostic potential.instead of (normal, inflamed, and infected mice with E. coli to assess thetracer’s diagnostic potential.

## Experimental

### Materials and methods

All chemicals utilized in this study were of analytical grade. Cefaclor, with the chemical formula C_15_H_14_ClN_3_O_4_S and a molecular weight of 367.8 gm/mol, was procured from the Pharmaceutical Chemicals Company, Egypt, and employed without further purification. Acetone served as a solvent and was also used without additional purification. Double-distilled water was used for all experimental procedures, and all chemicals used to prepare the different pH media, ranging from 2 to 11, were purchased from Merck. Chloramine-T [*N*-chloro-*p*-toluene sulfonamide sodium salt (CAT)] and *N-N*-bromosuccinamide (NBS) were sourced from Aldrich, while Iodogen (1,3,4,6-tetrachloro-3α,6α-diphenylglycouril) was obtained from Pierce Chemical Company. Thin-layer chromatography (TLC) aluminum sheets (20 × 25 cm) SG-6 F_254_ were supplied by Merck. Na^131^I (5 mCi/mL) in NaOH was obtained from the Radioisotope Production Factory (RPF) of the Egyptian Atomic Energy Authority.

### Equipment

Radioactivity measurements were conducted utilizing a calibrated gamma counter (Nucleus Model 2010), which is equipped with a well-type sodium iodide crystal doped with thallium [NaI(Tl)], ensuring high sensitivity for gamma emissions, particularly those of iodine-131. The system underwent routine validation for energy resolution and counting efficiency, with background correction applied before each measurement. Assessments of radiochemical purity, stability, and purity were performed using TLC and high-performance liquid chromatography (HPLC). The system included a solvent delivery module (S 2100, Sykam, Germany), an injector valve bracket (S 5111, Sykam, Germany) fitted with a 20 µL sample loop, and a UV/Vis multichannel detector (S 3240, Sykam, Germany). Separation was achieved using a Lichrosorb RP-18 reversed-phase column (Serial No. 15172204), optimized for hydrophobic interactions and retention of iodinated compounds. The entire system was operated under the Chroma Star Light 6.0 software, which facilitated real-time chromatogram acquisition, peak integration, and automated data export.

### Animal models

The biodistribution study was conducted using adult male Swiss Albino mice (age: 6 weeks), each weighing between 25 and 35 grams. The animals were obtained from the Biological Applications Department of the Nuclear Research Center, Egyptian Atomic Energy Authority. All mice were maintained under standardized laboratory conditions in a clean-air facility, with controlled environmental parameters including a relative humidity of 60 ± 4%, ambient temperature of 24 ± 5 °C, and a regulated 12-hour light/dark cycle. Throughout the experimental period, animals were provided with unrestricted access to standard rodent chow and filtered drinking water to ensure adequate nutrition and hydration.

### Ethical approval

Ethical approval was obtained from the Research Ethics Committee of the National Center for Radiation and Technology (RECNCRRT), Cairo, Egypt (Approval No. F/3A/2025). All animal experiments were conducted under EAEA guidelines, with anesthesia (xylazine/ketamine) and humane euthanasia by cervical dislocation to ensure compliance with ethical standards.

### Induction of inflammation

To evaluate the biodistribution responses elicited by infection and inflammation, adult male Swiss Albino mice were randomly assigned to three experimental groups (*n* = 7 per group). Group I served as the untreated control. Group II was designated for bacterial infection and injected intramuscularly with 200 µL of an *Escherichia coli* suspension, containing about 2 × 10^8^ colony-forming units (CFU), into the left lateral thigh muscle [22]. The *E. coli* strain was sourced from the Biological Applications Department at the Nuclear Research Center of the Egyptian Atomic Energy Authority. Post-inoculation, the animals were monitored for 48 hours to verify infection establishment until marked swelling appeared at the injection site. Group III underwent induction of sterile inflammation via intramuscular injection of 200 µL of autoclaved turpentine oil into the left lateral thigh muscle [23]. This intervention provoked a localized inflammatory response, characterized by observable swelling at the injection site within 48 hours.

### Radiolabeling of cefaclor with Na^131^I

The radiolabeling procedure was conducted in a 3 mL screw-capped reaction vial. Oxidizing agents, including CAT, iodogen, iodogen with glass frits, or NBS (50–300 μg), were precipitated onto the inner wall of the vial to maximize the reactive surface area. Cefaclor (25–150 μg), predissolved in acetone at a concentration of 1 mg/mL, was subsequently added, followed by 10 μL of Na^131^I solution (specific activity: 0.014 ± 0.001 GBq/μmol). The reaction mixture was supplemented with 100 μL of buffer to attain the targeted pH levels (2, 4, 6, 7, 9, or 11), and 100 μL of solvent (acetonitrile, acetone, ethanol, dimethylsulfoxide [DMSO], or dimethylformamide [DMF]) was incorporated to assess solvent influences. The mixture was incubated at controlled temperatures ranging from 25 to 100 °C for specified durations. The reaction was quenched by the addition of 50 μL of 0.1 N sodium metabisulfite [24]. The total volume of the vial contents during the labeling process is about 0.36 to 0.46 mL, depending on the reagents and solvents used. All experimental parameters were evaluated in triplicate to ensure reproducibility and statistical robustness. Gamma counter measurements were adjusted by subtracting background radiation before data analysis. Radiolabeling efficiency was measured by TLC, with radioactivity quantified using a γ-scintillation counter [25–27].

### Lipophilicity study

Lipophilicity (logP) values were estimated using the Swiss ADME predictive tool, applying the Crippen method to ensure consistency with standard ADME predictions [28]. The chemical structures of both unmodified cefaclor and the iodinated derivative ([^131^I] cefaclor) were entered, with the iodine atom assumed to be substituted at the para (4-) position of the benzene ring. This site was selected because of the activating effect of the amino group, making it the most reactive position for electrophilic substitution [29, 30].

### Invitro stability of [^131^I]Cefa in serum

The stability of [^131^I]Cefa was assessed in vitro by incubating an aliquot (0.1 mL) of the final preparation in freshly collected human serum (0.9 mL) at 37 °C for 24 hours. Radiolabeling efficiency was monitored at defined intervals using TLC.

### Purification of [^131^I]labeled cefaclor

Radiolabeling efficiency of [^131^I]labeled cefaclor was assessed by TLC and confirmed using HPLC.

### TLC analysis

TLCwas employed to evaluate the radiolabeling purity of the synthesized [^131^I]-labeled cefaclor, [^131^I]Cefa. A specific aliquot of the radiolabeled compound was precisely applied to the origin of a silica gel TLC strip (approximately 14 cm in length) using a calibrated micropipette. The chromatographic process commenced by vertically immersing the strip into a pre-prepared mobile phase consisting of methylene chloride and ethyl acetate in a 2:1 (v/v) volumetric ratio. Upon the solvent front reaching an optimal distance, the strip was removed from the chamber, thoroughly air-dried, and subsequently segmented into uniform 1 cm sections [31]. Each segment was individually analyzed for radioactivity using a sodium iodide (NaI) well-type scintillation detector to quantify the distribution of radiolabeled species. The chromatographic profile revealed that free iodide (^131^I^−^) remained localized near the origin, exhibiting a retention factor (Rf) in the range of 0.0–0.1. In contrast, the ^131^I-labeled cefaclor, [^131^I]Cefa, migrated with the solvent front, displaying an R_f_ value between 0.8 and 1.0, indicative of successful labeling and separation from unbound radioiodine.

### HPLC analysis

The radiolabeling efficiency of the synthesized compound [^131^I]Cefa was quantitatively evaluated using analytical high-performance liquid chromatography (HPLC). A 20 µL aliquot of cefaclor and the reaction mixture were separately injected into the HPLC system. Chromatographic separation was conducted on a reverse-phase C18 column (250 mm × 4.6 mm, particle size 5 µm). The mobile phase comprised a phosphate buffer (pH 3.4) and acetonitrile in a volumetric ratio of 80:20 (v/v), delivered at a constant flow rate of 0.5 mL/min [32]. Detection was performed using a UV spectrophotometer set at 254 nm wavelength. Cefaclor was separately injected and eluted to attain its retention time. Then, in another run to determine the retention time of iodine-131 and ^131^I-labeled cefaclor, fractions corresponding to the [^131^I]Cefa were collected using an automated fraction collector. The collected fractions were subsequently subjected to evaporation under reduced pressure. The dried residues were reconstituted in sterile saline solution and passed through a 0.22 µm Millipore membrane filter to ensure sterility. The radioactivity of the purified [^131^I]Cefa was measured using a sodium iodide (NaI(Tl)) scintillation crystal coupled to a single-channel analyzer for gamma detection.

### Modeling insights into [^131^I]Cefa stability

Theoretical approaches have become indispensable in the rational design of radiopharmaceutical agents, offering predictive insights into molecular behavior before experimental synthesis. This study examines the structural stability of [^131^I]Cefa through semi-empirical modeling techniques to determine the most energetically favorable coordination pattern. Molecular structures representing various binding modes between cefaclor and radioactive iodine-131 were constructed and energy-minimized using the PM3 method within Spartan software (Wavefunction Inc., Irvine, CA). This platform facilitates efficient molecular modeling and descriptor analysis, enabling the evaluation of thermodynamic parameters. The PM3 basis set was selected for its computational efficiency and demonstrated accuracy in modeling organo iodine systems. Each complex was optimized to its lowest energy conformation, and the total electronic energy values were recorded in atomic units (a.u.) for comparative analysis, revealing the most stable configuration among the proposed models [33].

### Biological evaluation studies

In vivo biological evaluation studies were conducted to assess the uptake and targeting efficiency of the [^131^I]Cefa tracer in Swiss Albino male mice (25–35 g). The experimental design comprised three distinct groups: control (healthy), infected (*E. coli-induced*), and inflamed (sterile inflammation induced by turpentine oil). Each group was further divided into four time-point cohorts: 30, 60, 120, and 240 minutes post-injection, with seven mice per subgroup (*n* = 7), ensuring statistical robustness and reproducibility across biological replicates. Each animal received an intravenous administration of 0.2 mL of [^131^I]Cefa solution (7.2 MBq). At each specified time interval, mice were anesthetized with an intravenous injection of xylazine (5 mg/kg) and ketamine (60 mg/kg), following established protocols [34]. Adequate anesthesia was confirmed by the absence of the pedal withdrawal reflex, ensuring minimization of pain during handling and inflammation induction. Once anesthesia was achieved, animals were positioned supine and euthanized by cervical dislocation, a method recognized as humane and effective for ensuring rapid death under ethically approved conditions. Each organ and tissue was excised with precision, rinsed with isotonic saline, blotted dry, weighed, and subsequently measured for radioactivity using a NaI(Tl) γ-ray scintillation counter [35]. The percentage of injected dose per organ (%ID/organ) was calculated after subtracting the background with physiological assumptions for blood, bone, and muscle masses estimated at 7%, 10%, and 40% of total body weight, respectively [36].

### Data analysis

All data were analyzed using one-way ANOVA, with results expressed as mean ± standard error of the mean (SD). Statistical significance was set at *p* < 0.05.

## Results and discussion

Cefaclor was successfully radiolabeled with Na^131^I to establish a reproducible method for generating a radiopharmaceutical candidate suitable for biological evaluation of cefaclor. The radioiodination proceeded via an electrophilic substitution mechanism, in which the [^131^I]^+^ electrophile preferentially attacked the electron-rich aromatic ring of the phenylglycine side chain. The activating effect of the amino group facilitated substitution at the para (4-) position of the benzene ring relative to the aminoacetamido substituent, which represents the most reactive site for electrophilic attack within this molecular scaffold [37]. This regioselectivity is consistent with the electronic characteristics of the cefaclor structure and supports the feasibility of the targeted radioiodination.

To maximize the radiolabeling efficiency, all the reaction parameters were optimized sequentially in a systematic stepwise manner. Each factor, including the amount of cefaclor (25–150 μg), type and concentration of oxidizing agents (CAT, iodogen, iodogen with glass frits, or NBS at 50–300 μg), pH of the reaction medium (2–11), incubation temperature (25–100 °C), reaction time (5–60 min), and solvent type (acetonitrile, acetone, ethanol, DMSO, and DMF), was investigated individually. For each parameter, the condition yielding the highest radiochemical yield was selected and fixed before proceeding to the next variable was selected and fixed. All experiments were performed in triplicate, and the yields were determined by TLC and quantified using γ-scintillation counting after background correction. Through this sequential optimization, the optimal conditions were identified in the presence of 100 μg of iodogen deposited on glass frits. Cefaclor (100 μg) was radiolabeled with Na^131^I via electrophilic substitution, achieving a radiolabeling efficiency of 90 ± 0.56% in a neutral medium (pH 7) at 60 °C for 20 min. These findings confirm the success of the developed protocol and highlight its potential utility for subsequent biological evaluation studies.

### Factors affecting radiolabeling efficiency of [^131^I]Cefa

#### Effect of cefaclor amount

**Fig. 2:**
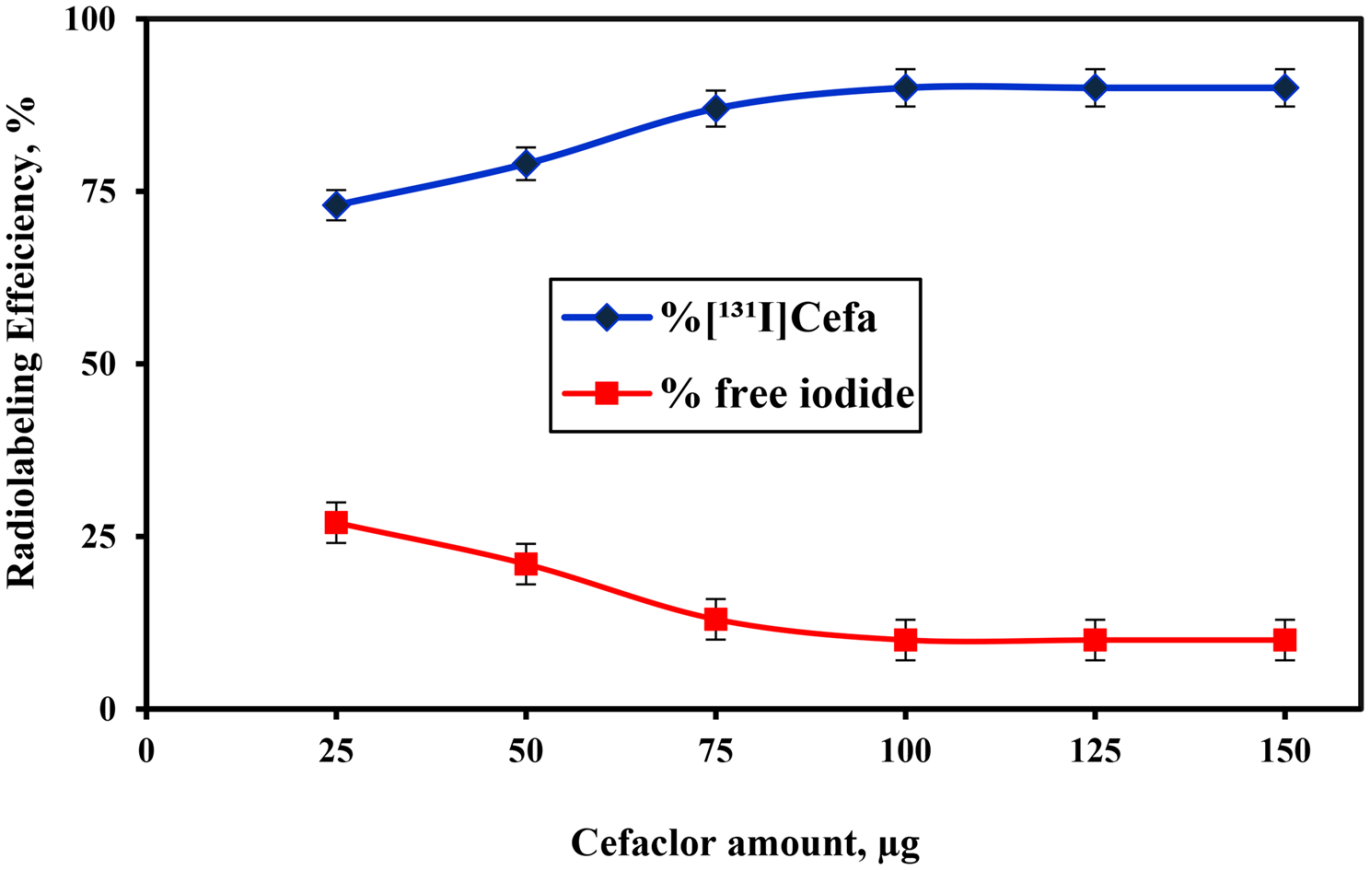
Variation in the radiolabeling efficiency of [^131^I]Cefa as a function of Cefa amount (25–150) µg: [25–150 µg of Cefa + 100 µg of iodogen deposited on glass frit+ 100 µl of pH 7 + 10 µL of Na^131^I(0.014±0.001 GBq/µmol)] at 60 °C for 20 min

The influence of varying amounts of cefaclor on the radiolabeling efficiency of radioiodinated cefaclor [^131^I]Cefa was systematically examined under controlled reaction conditions. As depicted in Fig. 2, cefaclor was incrementally added in amounts ranging from 25 to 150 µg to a constant amount of iodogen (100 µg), which had been pre-deposited on a glass frit serving as the oxidizing agent. The reaction was conducted at a neutral pH (pH 7) and maintained at 60 °C for 20 minutes to ensure optimal labeling kinetics. A progressive increase in the radiolabeling efficiency was observed with rising cefaclor concentration, from 72.7 ± 0.42% at 25 µg to a maximum of 90 ± 0.56% at 100 µg. This trend suggests that the availability of cefaclor plays a critical role in facilitating efficient electrophilic substitution ofiodine-131, likely by improving the stoichiometric balance between the substrate and the oxidizing agent. The observed plateau beyond 100 µg indicates that the system had reached a saturation threshold, beyond which additional cefaclor did not contribute to further yield improvement [38].

### Effect of pH

**Fig. 3:**
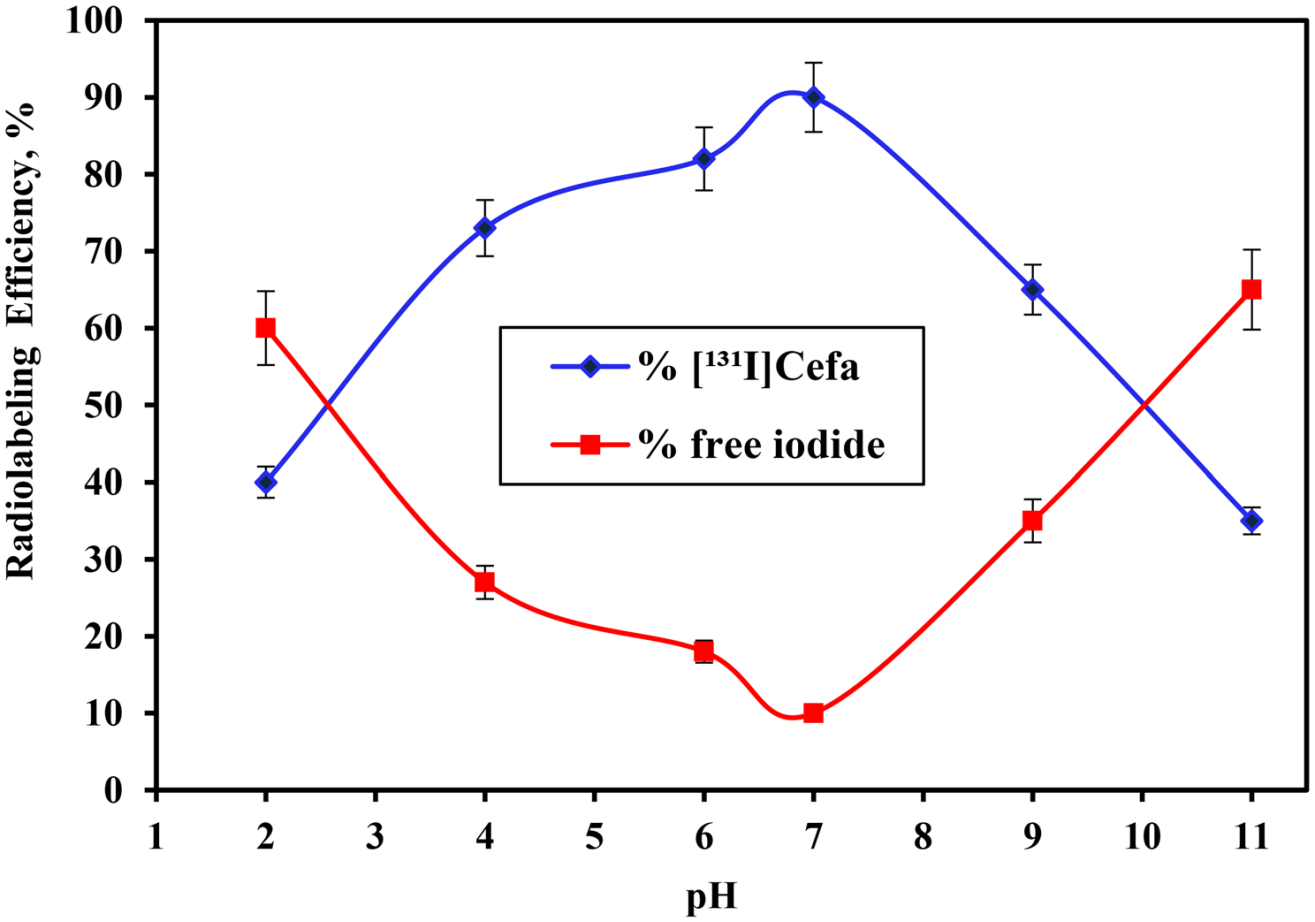
Variation in the radiolabeling efficiency of [^131^I]Cefa as a function of pH (2–11) [100 µg Cefa + 100 µg of iodogen deposited on glass frit +100 μL buffer at various pH (2–11) +10 µL of Na^131^I (0.014±0.001 GBq/µmol) +] for 20 min at 60 °C

The radiolabeling efficiency of cefaclor was observed to be significantly dependent on pH, as illustrated in Fig. 3. An optimal radiolabeling efficiency of 90 ± 0.32% for [^131^I]Cefa was attained at pH 7, aligning with the established chemical stability of cefaclor under neutral conditions. This enhanced yield is attributed to the favorable protonation of the aromatic ring at neutral pH, which facilitates the formation of a reactive intermediate (Ar–H^+^) that readily undergoes electrophilic substitution by the iodonium ion (I^+^), thereby promoting efficient labeling [39]. Conversely, shifting the reaction medium towards acidic conditions resulted in a substantial decrease in radiolabeling efficiency, reaching only 40 ± 0.22% at pH 2. This reduction is likely due to the predominance of iodine monochloride (ICl) species under acidic conditions, which possess a lower oxidation potential compared to hypochlorous acid (HOCl), thereby diminishing the efficiency of the iodination process [40]. Similarly, in alkaline media, at pH 11, the radiolabeling efficiency was notably reduced to 35 ± 0.34%. This decline is attributed to the decreased concentration of hypoiodous acid (HOI), the key electrophilic species responsible for aromatic substitution reactions in radioiodination [41]. The diminished availability of HOI under basic conditions compromises the formation of [^131^I]Cefa. underscoring the critical role of pH in modulating the speciation and reactivity of iodine intermediates. These findings collectively underscore the necessity of maintaining a neutral pH environment to optimize the radioiodination of cefaclor, ensuring both chemical stability and maximal electrophilic substitution efficiency.

### Effect of different oxidizing agents

**Fig. 4:**
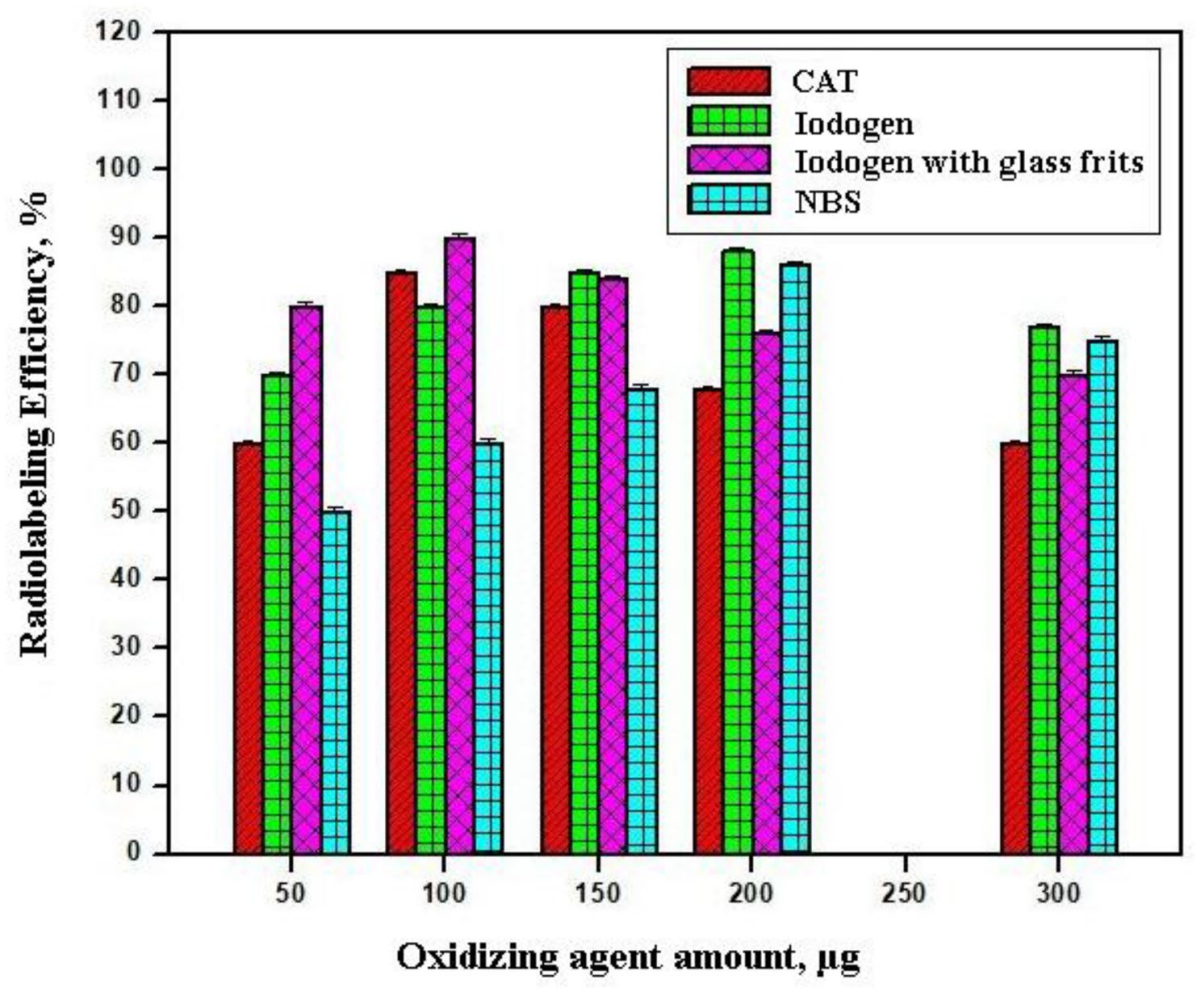
Variation in the radiolabeling efficiency of [^131^I]Cefa as a function of different amounts of oxidizing agents (50–300 µg): [100 µg of Cefa + (50–300) µg of various oxidizing agents + 100 µL of pH 7 + 10 µL of Na^131^I (0.014±0.001 GBq/µmol)] at 60 °C for 20 min

The efficiency of radioiodination reactions is critically dependent on both the type and concentration of the oxidizing agents, as shown in Fig. 4. In this study, the comparative performance of CAT, iodogen (free and immobilized on glass frits), and NBS was systematically evaluated for the radiolabeling of cefaclor under standardized conditions (pH 7, 60 °C, 20 min). CAT is a widely used oxidizing agent in radioiodination due to its ability to generate reactive iodine species under mild conditions. Mechanistically, CAT facilitates the oxidation of iodide (I^−^) to the electrophilic iodonium ion (I^+^) via hypochlorite intermediates [42]. This iodonium ion (I^+^) species subsequently participates in electrophilic substitution on the aromatic ring of the substrate. At a concentration of 100 µg, CAT yielded a moderate radiolabeling efficiency of 85 ± 1.2%. However, increasing CAT concentrations resulted in a progressive decline in efficiency. This reduction can be attributed to the excessive generation of oxidative species, which not only promote iodination but also induce non-specific side reactions such as chlorination and structural degradation of cefaclor. These findings emphasize that while CAT is effective at moderate levels, its overuse compromises labeling efficiency due to uncontrolled oxidation.

Iodogen, another commonly employed oxidizing agent, operates through a distinct mechanism. It oxidizes iodide to iodonium ion (I^+^) via electron transfer at its aromatic ring, thereby providing a controlled release of reactive iodine species [43]. Unlike CAT, iodogen is less aggressive, which minimizes non-specific oxidation and favors selective iodination. A dose-dependent improvement in radiolabeling efficiency was observed, with yields increasing from suboptimal levels at 50–150 µg to a maximum of 88 ± 1.1% at 200 µg. Importantly, when iodogen was immobilized on glass frits, the efficiency peaked at 100 µg, achieving 90 ± 0.85%, a result comparable to that obtained with 200 µg of free iodogen. This enhanced performance can be explained by the increased surface area and controlled release of oxidizing species provided by the frit support, which improves reaction kinetics and reduces side reactions. Thus, the iodogenglass frit system represents a more efficient and economical approach, achieving high radiolabeling yields with reduced reagent consumption [44].

In contrast, NBS exhibited the least favorable performance among the tested oxidants. Even at the highest concentration (200 µg), the radiolabeling efficiency did not exceed 86 ± 1.1%. This limited efficacy may be due to the relatively lower oxidative potential of NBS or its inability to generate sufficient I^+^ species under the applied reaction conditions. These findings underscore the importance of mechanistic understanding in optimizing radioiodination protocols and suggest that immobilized iodogen offers a promising strategy for achieving efficient and controlled radiolabeling of cefaclor.

### Effect of reaction temperature

**Fig. 5:**
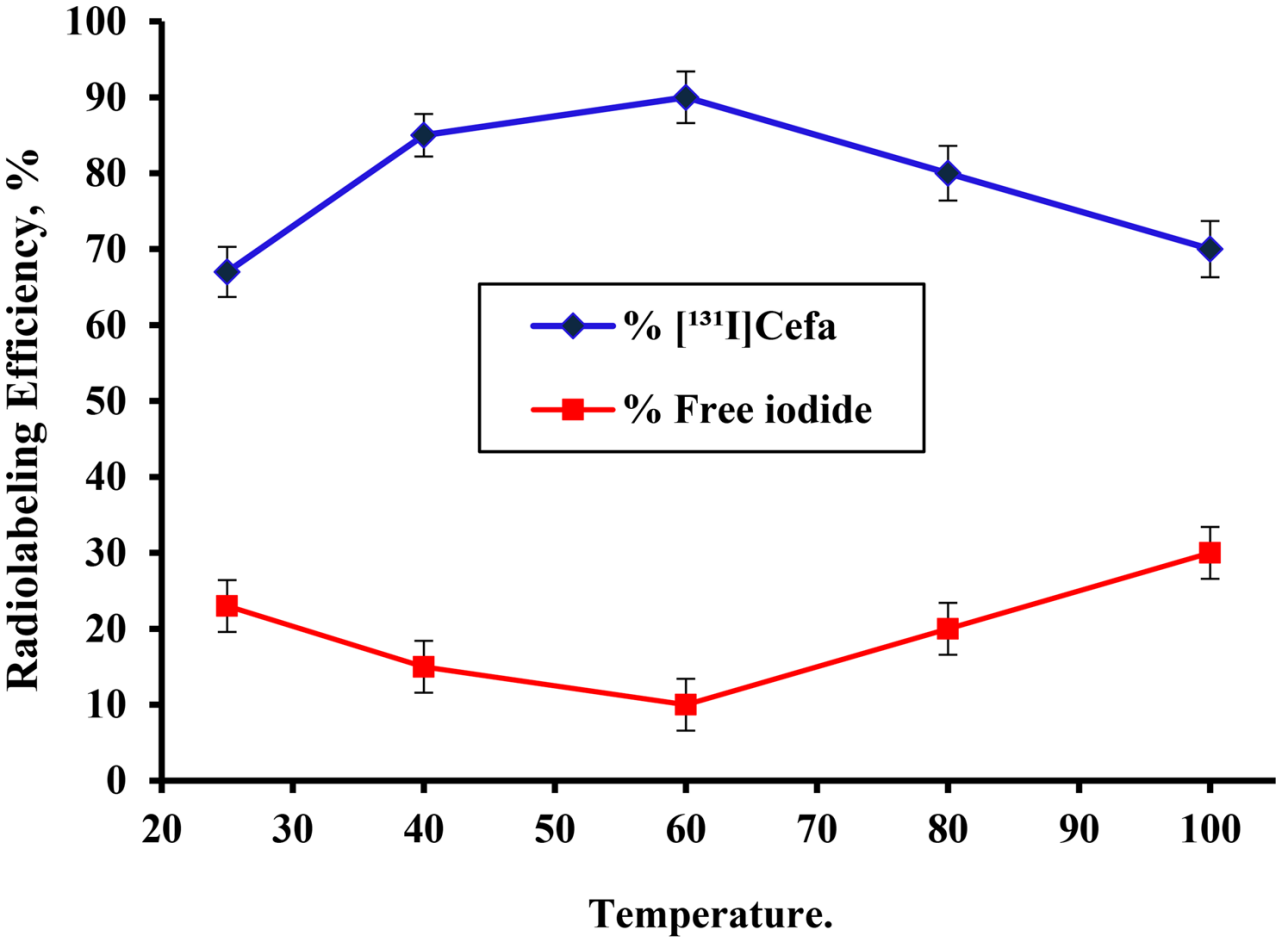
Variation in the radiolabeling efficiency of [^131^I]Cefa as a function of various temperatures (25–100 ° C):[100 µg of Cefa +100 µg of iodogen deposited on glass frits + 100 µL of pH 7 + 10 µL of Na^131^I (0.014±0.001 GBq/µmol)] at (25–100 ° C) for 20 min

Reaction temperature is a critical parameter in radiolabeling protocols, as it directly influences the kinetics of electrophilic substitution and the stability of both the substrate and the reactive intermediates. As illustrated in Fig. 5, the radiolabeling efficiency of [^131^I]Cefa was significantly affected by temperature variations under standardized reaction conditions (pH 7, 20 min). At ambient temperature (25°C), the radiolabeling efficiency was relatively low, achieving only 67 ± 1.3%. This modest yield may be attributed to insufficient thermal energy to facilitate the oxidation of iodide (I^−^) to the reactive iodonium ion (I^+^), thereby limiting the extent of labeling [45]. Upon gradual elevation of the reaction temperature to 40 °C and subsequently to 60 °C, a notable improvement in radiolabeling efficiency was observed, increasing from 85 ± 1.2% to a maximum of 90 ± 1.1%, respectively. This enhancement reflects the favorable impact of moderate thermal activation on reaction kinetics, facilitating efficient generation of electrophilic iodine species and promoting substitution at the aromatic ring of cefaclor. However, further increase in temperature to 100 °C resulted in a pronounced decline in radiolabeling efficiency, dropping to 70 ± 1.5%. This reduction is likely due to thermal degradation of the labeled compound or decomposition of the oxidizing agent, which compromises the availability of active iodine species [46]. Elevated temperatures may also accelerate non-specific side reactions, including hydrolysis or structural denaturation of cefaclor, thereby diminishing labeling efficiency. These findings underscore the importance of optimizing reaction temperature to balance activation energy with molecular stability. A temperature of 60 °C appears to offer the most favorable conditions for achieving high radiolabeling efficiency without compromising the integrity of the labeled product.

### Effect of reaction time

**Fig. 6:**
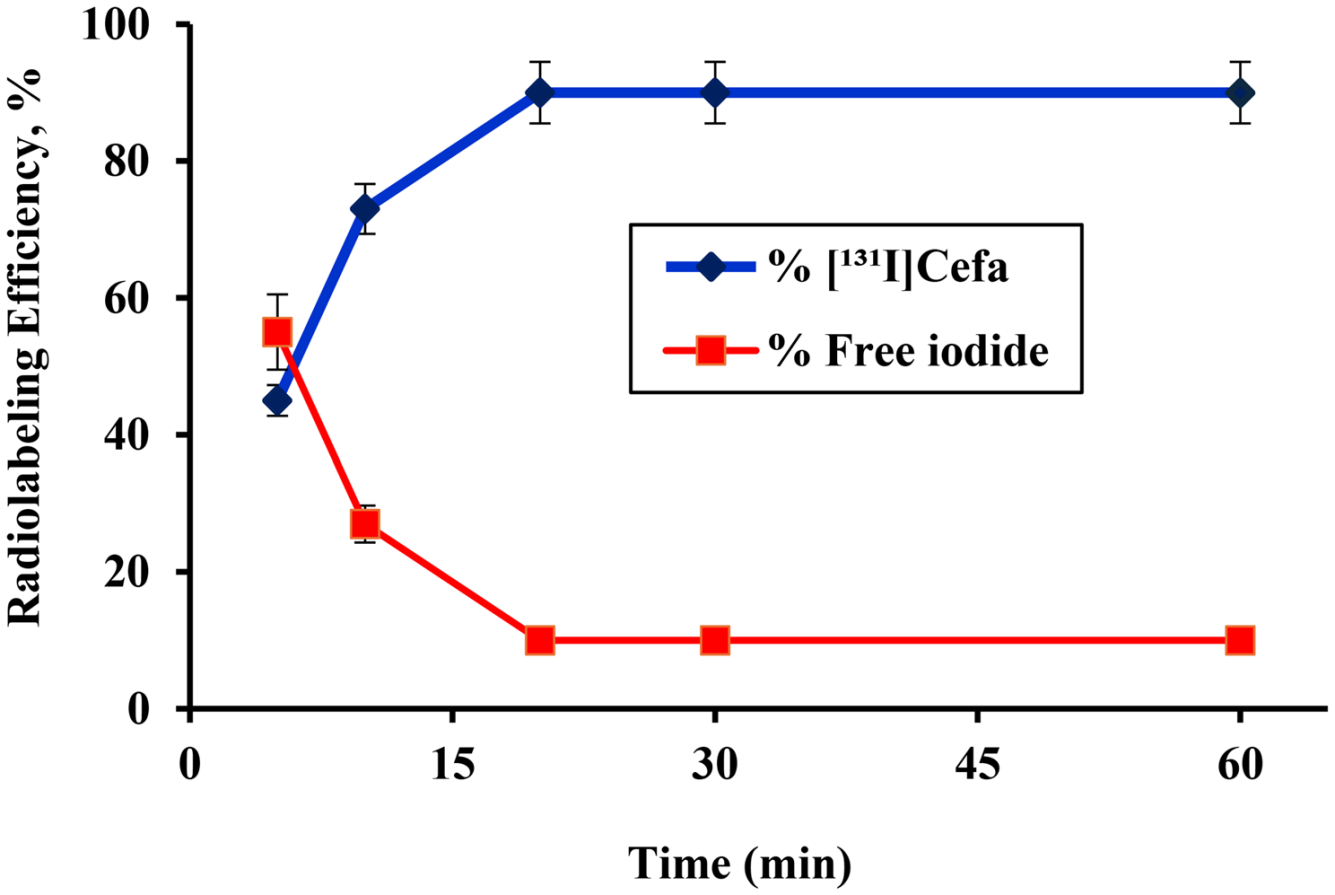
Variation in the radiolabeling efficiency of [^131^I]Cefa as a function of reaction time (5–60 min):[100 µg of Cefa +100 µg iodogen deposited on glass frits + 100 µL pH 7 + 10 µL of Na^131^I (0.014±0.001 GBq/µmol)] at 60 °C, for (5–60) min

The impact of reaction time on the radiolabeling efficiency of [^131^I]Cefa was systematically assessed over a duration ranging from 5 to 60 minutes, as depicted in Fig. 6. The data indicate a rapid and proportional increase in radiolabeling efficiency during the initial phase of the reaction, with a pronounced rise observed between 5 and 20 minutes. At 20 minutes, the radiolabeling efficiency reached its maximum of approximately 90 ± 1.2%, indicating the completion of the labeling process under the optimized reaction conditions. Beyond the 20-minute mark, the radiolabeling efficiency plateaued and remained stable up to 60 minutes, suggesting that the reaction had reached equilibrium and that further extension of the reaction time did not confer any additional benefit. This stabilization reflects the efficient consumption of available iodide and the saturation of reactive sites on the cefaclor molecule, confirming that 20 minutes is sufficient to achieve maximal labeling efficiency.

### Effect of solvent on radiochemical efficiency of [^131^I]Cefa

The impact of solvent selection on the radiolabeling efficiency of [^131^I]Cefa was systematically assessed using Cefaclor (100 μg) dissolved in various organic solvents in the presence of 100 µg of iodogen deposited on glass frits as an oxidizing agent. As detailed in Table 1, the radiolabeling efficiency exhibited significant variation contingent upon the solvent utilized, underscoring the pivotal role of solvent polarity and solubilizing capacity in optimizing labeling efficiency. Among the solvents evaluated, acetone achieved the highest radiolabeling efficiency (90 ± 0.8%), indicating superior compatibility with the iodination reaction conditions and enhanced solubility of Cefaclor. Ethanol also demonstrated a relatively high yield (85 ± 0.2%), likely attributable to its moderate polarity and capacity to facilitate efficient electrophilic substitution [47]. Conversely, DMF and acetonitrile yielded lower results (73 ± 0.1% and 75 ± 0.3%, respectively), which may be attributed to suboptimal solvation dynamics or potential interference with the labeling process. DMSO exhibited a slightly improved yield (77 ± 0.2%) compared to DMF, possibly due to its higher dielectric constant and superior stabilization of reaction intermediates. Overall, the data suggest that acetone provides the most favorable environment for radiolabeling Cefaclor with iodine-131, likely due to its balanced polarity, low nucleophilicity, and efficient substrate dissolution, thereby maximizing the incorporation of the radioisotope.

**Table 1:** Effect of various solvents upon the radiolabeling efficiencyof [^131^I]Cefaclor

Solvent	Radiochemical efficiency (%)
DMF	73 ± 0.1%
DMSO	77 ± 0.2%
Ethanol	85 ± 0.2%
Acetone	90 ± 0.8%
Acetonitrile	75 ± 0.3%

### Lipophilicity study

We estimated logP values using the Swiss ADME predictive tool (employing the Crippen method for consistency with standard ADME predictions). For unmodified cefaclor (the non-radiolabeled analog), the estimated logP was 0.62. For the iodinated derivative [^131^I] cefaclor, with iodine substituted at the para (4-) position of the benzene ring owing to the activating effect of the amino group, making this the most reactive site for electrophilic attack, the estimated logP is 1.23. This increase of approximately 0.61 logP units reflects the hydrophobic contribution of the iodine atom on the aromatic ring, rendering the radiolabeled compound moderately more lipophilic. Both forms remain predominantly hydrophilic (logP < 2, which is characteristic of cephalosporins). The enhanced lipophilicity of [^131^I] cefaclor may slightly improve passive membrane permeation and tissue distribution in lipid-rich compartments, potentially benefiting its application in imaging or therapeutic contexts. However, this shift is unlikely to substantially alter the overall pharmacokinetics or other ADME properties, as the core scaffold remains intact.

### In-vitro stability of [^131^I]Cefa in serum

**Fig. 7:**
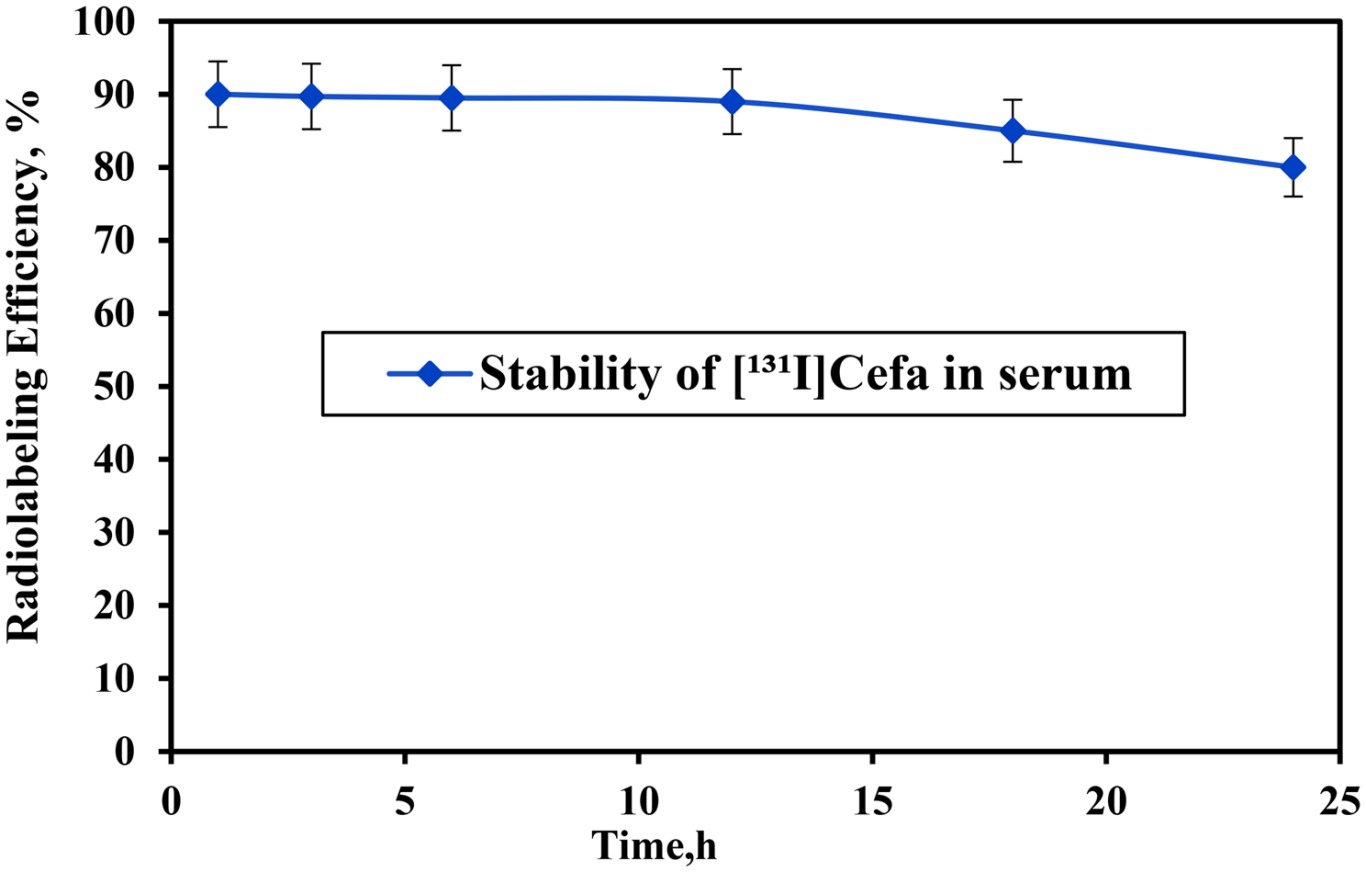
In-vitro stability of [^131^I]Cefa with fresh serum at various time intervals

The in vitro stability of the radiolabeled compound [^131^I]Cefa was systematically assessed inhuman serum to evaluate the integrity of the radiolabel over time (Fig. 7). The compound exhibited high initial stability, maintaining a radiochemical purity of 90 ± 0.2% at 1 hour and 89.5 ± 0.5% at 4 hours. This stability persisted with only minimal decline up to 12 hours (88 ± 0.8%), indicating strong resistance to deiodination and metabolic degradation within the serum matrix. Beyond this period, a gradual reduction in radiochemical purity was observed, with values decreasing to 85 ± 0.4% at 16 hours and 80 ± 0.3% at 24 hours. These findings demonstrate that [^131^I]Cefa retains substantial stability for at least 12 hours under physiological conditions, a property that is critical for its potential application as a radiotracer in diagnostic imaging and therapeutic interventions [48].

### Radiolabeling purity of [^131^I]Cefa

**Fig. 8:**
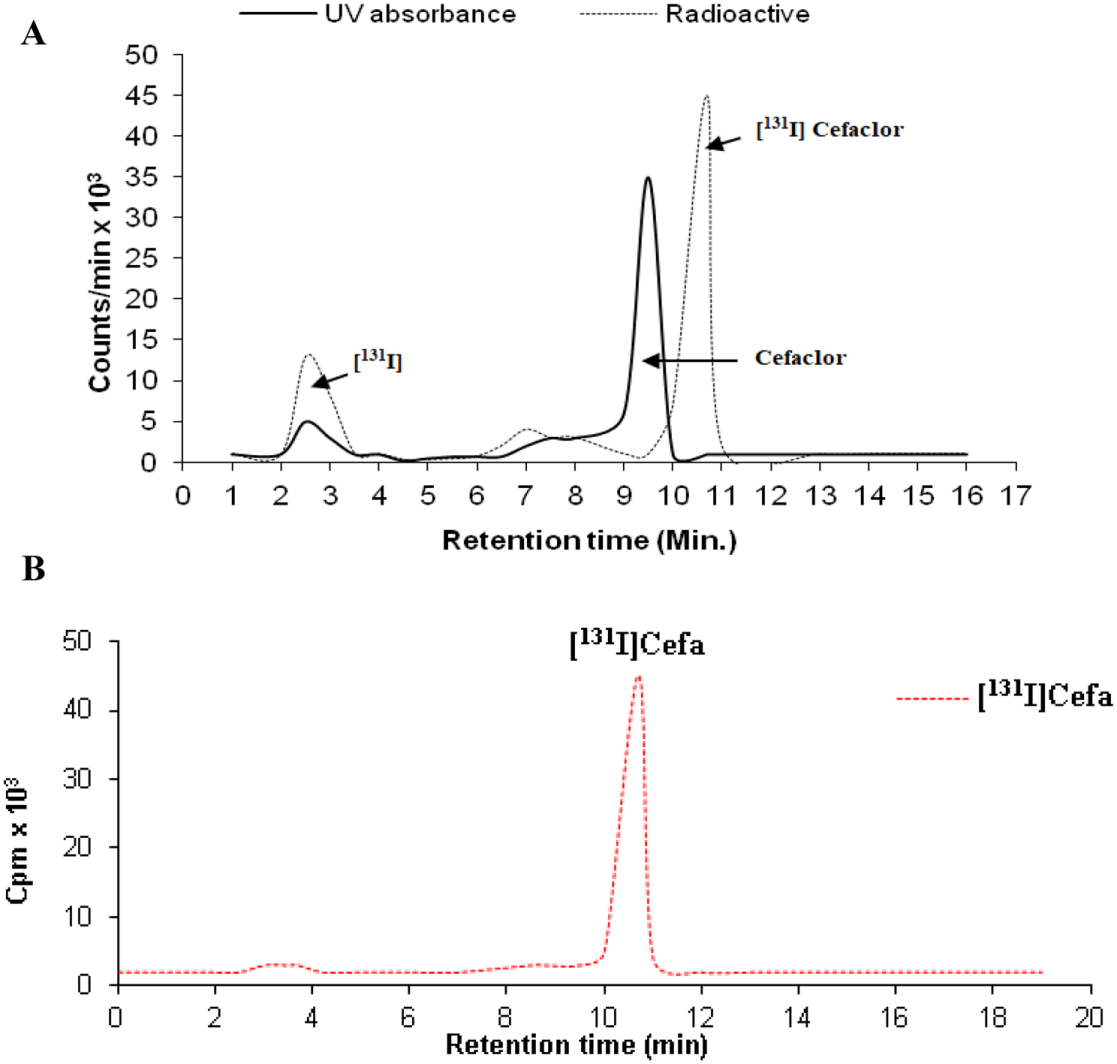
(**a**) Representative HPLC elution profile of native cefaclor and radiolabeled [^131^I]Cefaclor. (**b**) Representative HPLC elution profile of radiolabeled [^131^I]Cefa

The chromatographic separation of [^131^I]Cefa from its unlabeled portion was accomplished using HPLC, as illustrated in Fig. 8a. The elution profile revealed distinct retention times for each component, confirming the effectiveness of the purification protocol. Notably, [^131^I]Cefa displayed a retention time of 10.7 minutes, while native cefaclor eluted at 9.5 minutes. Additionally, free radioiodide was detected at an earlier retention time of 2.5 minutes, demonstrating successful isolation of the radiolabeled compound from unbound iodine. The fractions corresponding to [^131^I]Cefa were collected and subjected to evaporation under reduced pressure to remove residual solvents. The resultant dried radiolabeled compound was then reconstituted in sterile saline solution and passed through a 0.22 µm Millipore membrane filter under aseptic conditions to guarantee sterility and appropriateness for subsequent biological applications. The purified [^131^I]Cefa exhibited a molar activity of 0.014 ± 0.001 GBq/µmol, ensuring high sensitivity for the detection of inflammatory foci. These results emphasize the reliability of the HPLC approach for achieving high radiochemical purity and selective isolation of [^131^I]Cefa, which is essential for subsequentpharmacological or biological assessments [49]. To verify the purity and structural integrity of [^131^I]Cefa, the radioligand was reinjected following elution and isolation (Fig. 8b). As anticipated, the ^131^I-labeled cefaclor eluted at 10.7 minutes, with a radiochemical purity ≥ 98% consistent with prior observations, and exhibited no detectable impurities.

### Computational modeling of [^131^I]Cefa coordination

Recent advancements in computational chemistry have markedly improved the rational design of radiopharmaceutical agents, particularly through the application of molecular descriptors and semiempirical modeling techniques. This study presents a PM3-based theoretical evaluation of [^131^I] iodocefaclor [^131^I]cefa tracers, revealing distinct coordination patterns with varying degrees of thermodynamic stability. Among the four proposed structures (a–d), complex (c) demonstrated the lowest total energy value (E = −8783.58180639 a.u.), indicating its superior stability compared to the other configurations, as shown in the Table 2. This finding suggests that the iodine atom in complex (c) adopts a spatial orientation and electronic environment that minimizes steric hindrance and optimizes the intramolecular interactions [50]. These results underscore the utility of semi-empirical methods in the preclinical screening of radiolabeled drug candidates and provide a predictive framework for further experimental validation.

**Table 2:** Optimized structures and energies of [^131^I]Cefa models

Descriptor	Total Energy (a.u)	Visual Configurations
Complex (a)	−1893.61811184	
Complex (b)	−8783.58169726	
Complex (c)	−8783.58180639	
Complex (d)	−8783.57406762

**Table 3:** The binding energy score of cefaclor and ^131^I-labeledcefaclor

Cefaclor		^131^I-labeledcefaclor	
S	RMSD	S	RMSD
**−7.248**	**1.291**	**−7.526**	**2.174**
−6.979	1.280	−7.278	2.174
−6.945	1.864	−7.252	1.121
−6.836	1.745	−7.246	1.528
−6.742	1.649	−7.176	2.178

### Molecular docking study

To elucidate the molecular interactions and binding affinities of cefaclor and its radiolabeled analog, ^131^I-labeled cefaclor, with *DNA Gyrase B*, molecular docking simulations were conducted using the Molecular Operating Environment (MOE) software. *Bacterial DNA gyrase B* (PDB ID: 3G7E) was selected as the target receptor because of its critical role in bacterial DNA replication and its established significance as an antimicrobial target [51]. Each ligand underwent docking analysis, generating ten plausible binding poses for each compound. The binding affinity of each pose was quantitatively assessed using the S-score, where lower values correspond to stronger interactions and more favorable binding conformations [52]. As presented in Table 3, ^131^I-labeled cefaclor exhibited a more favorable binding energy (−7.526 kcal/mol) with an RMSD of 2.174 Å compared to cefaclor (−7.248 kcal/mol, RMSD 1.291 Å), indicating the enhanced affinity of the radiolabeled derivative toward DNA gyrase B. This improvement in binding affinity may be attributed to the structural modifications introduced by radioiodination, which could enhance the molecular complementarity within the enzyme’s active site. Although the RMSD value of the best-scoring pose for ^131^I-labeled cefaclor was higher than that of the parent ligand, suggesting slightly lower conformational stability, the radiolabeled compound demonstrated a more favorable binding energy than the parent compound. The first five docking scores for both cefaclor and ^131^I-labeled cefaclor are presented in Table 3. Notably, the third-ranked pose of ^131^I-labeled cefaclor exhibited the lowest binding energy among all generated conformations, outperforming the corresponding cefaclor poses. These results collectively indicate that radioiodination enhances the binding potential of cefaclor to *DNA gyrase B*, supporting the potential therapeutic advantage of the radiolabeled derivative.

**Fig. 9:**
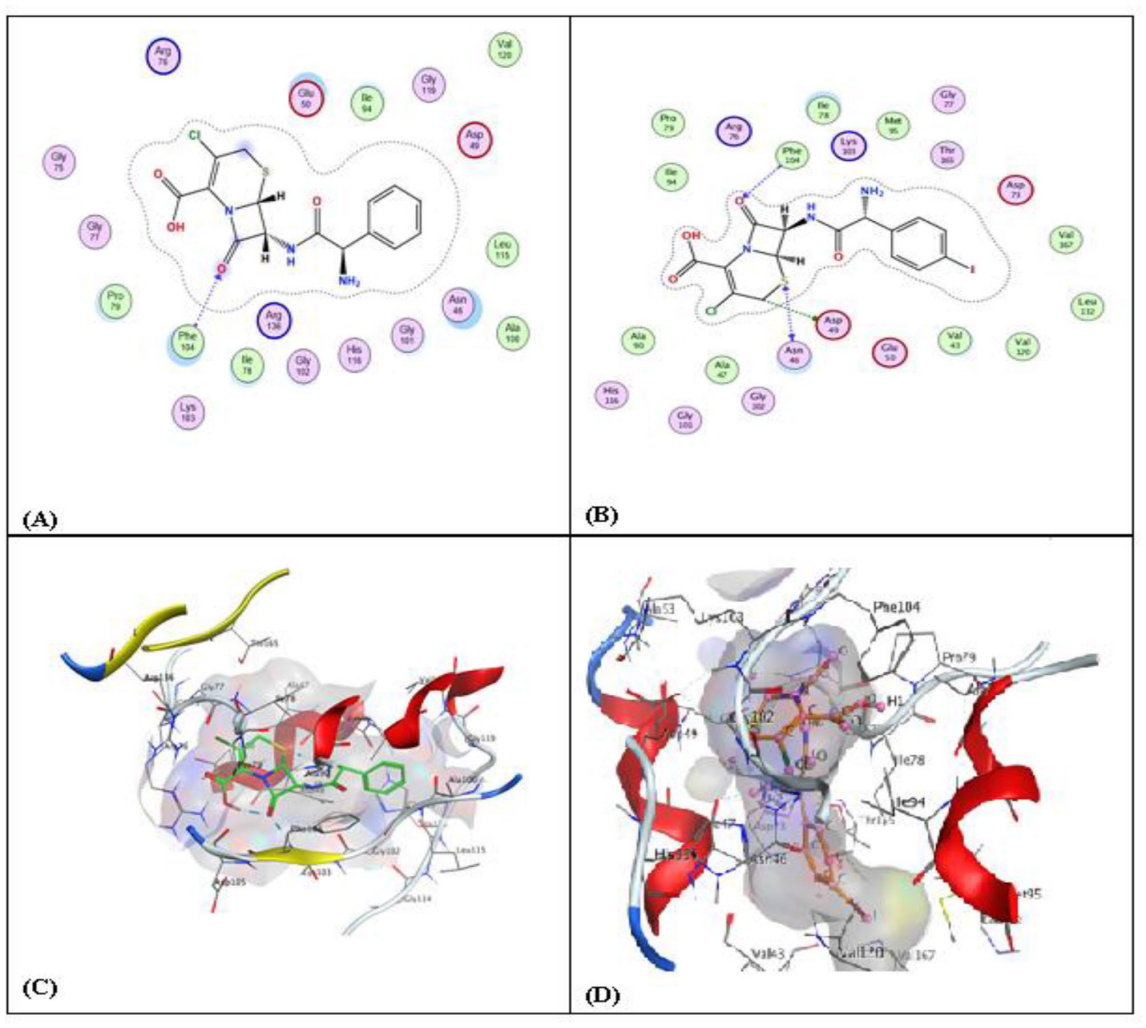
(**A-D**). Molecular interaction maps and surface representations of cefaclor and ^131^I-labeled cefaclor with DNA gyrase B (3G7E protein). (**A**) 2D interaction map of cefaclor with DNA gyrase B. (**B**) 2D interaction map of ^131^I-labeled cefaclor with DNA gyrase B. (**C**) 3D surface representation of cefaclor bound to DNA gyrase B. (**D**) 3D surface representation of ^131^I-labeled cefaclor bound to DNA gyrase B

Molecular docking analysis was conducted to examine and compare the binding characteristics of cefaclor and its radiolabeled analogue, ^131^I-labeled cefaclor, with *DNA Gyrase B* (PDB ID: 3G7E). The analysis of docking poses and ligand–protein interaction maps generated using MOE (Fig. 9A, C for cefaclor and Fig. 9B, D for ^131^I-labeledcefaclor) revealed distinct differences in their binding behavior within the active site. Cefaclor exhibited a relatively simple interaction profile, characterized by a single dominant hydrogen bond formed between the carbonyl oxygen atom (O_3_) of the ligand and the backbone amide nitrogen of PHE104, with an interaction distance of 3.11 Å and an interaction energy of −2.6 kcal/mol. This moderate but stable interaction suggests a favorable orientation of cefaclor within the ATP-binding pocket; however, the absence of additional stabilizing contacts indicates a limited binding network, which is consistent with its comparatively modest docking score [53]. In contrast, ^131^I-labeled cefaclor demonstrated a more extensive and diversified interaction pattern. The docking pose demonstrated that ^131^I-labeled cefaclor is accommodated deeply within the active site cavity, forming several hydrogen bonds with key residues. Notably, hydrogen bonding interactions were observed with Asp49 and Glu50, which are positioned within the conserved ATP-binding region. These acidic residues appear to stabilize the ligand via interactions with the amide and carboxylate functionalities of ^131^I-labeled cefaclor. Additional hydrogen bond interactions involving Asn46 and Lys103 further contribute to anchoring the ligand within the pocket. The involvement of Lys103 suggests electrostatic complementarity between the positively charged residue and the polar functionalities of the ligand, enhancing binding stability.

Moreover, hydrophobic interactions also play a significant role in stabilizing the ligand–protein complex. The aromatic ring of ^131^I-labeled cefaclor extends into a hydrophobic sub-pocket lined by residues such as Val43, Val120, Val167, Leu132, Ile78, and Met95. These van der Waals contacts likely contribute substantially to the overall binding affinity by promoting favorable hydrophobic packing. The presence of the iodine substituent on the aromatic ring may further enhance these interactions by increasing molecular polarizability and hydrophobic surface area. The iodine atom may also participate in halogen bonding interactions, although confirmation of such interactions would require further structural or computational validation. Collectively, these findings demonstrate that, unlike cefaclor, which relies primarily on a single hydrogen bond, ^131^I-labeled cefaclor establishes multiple stabilizing interactions, including dual hydrogen bonds and a distinctive halogen interaction. This more complex interaction network provides a plausible explanation for the superior docking score and predicted binding affinity of the radiolabeled analogue. Overall, the docking results indicate that incorporation of iodine-131 into the cefaclor structure enhances its molecular recognition and binding stability toward *DNA Gyrase B*, supporting the potential utility of ^131^I-labeled cefaclor as an improved radiolabeled antimicrobial candidate with enhanced receptor affinity.

### Biological evaluation studies of [^131^I]Cefa

The biodistribution study for the [^131^I]Cefa tracer in normal mice, as presented in Table 4, reveals a rapid and substantial systemic circulation, as demonstrated by the elevated blood uptake at 30 minutes post-injection (28 ± 1.4% injected dose [%ID]). This initial peak signifies efficient vascular distribution and implies swift absorption and dissemination of the radiolabeled compound. The ensuing reduction in blood radioactivity over time (25 ± 1.5%ID at 60 minutes, 10.5 ± 1.0%ID at 120 minutes, and 3.8 ± 1.2%ID at 240 minutes) reflects progressive clearance and redistribution to metabolizing and excretory organs, aligning with the established pharmacokinetics of cefaclor [54]. The kidneys, serving as the primary sites for metabolism and excretion, displayed distinct uptake patterns. Renal uptake was substantially higher, reaching a peak of 9.8 ± 1.3%ID at 60 minutes and sustaining elevated levels thereafter, consistent with cefaclor’s predominant renal elimination in its unaltered form. The pronounced renal uptake observed aligns with prior pharmacokinetic reports, which indicate that cefaclor is primarily excreted unchanged, with limited metabolic alteration. Consequently, this uptake likely represents renal elimination of the parent compound rather than metabolite accrual, obviating the need for supplementary in vivo metabolic stability assessments in this context [55]. The progressive augmentation in urinary excretion (from 8.0 ± 1.1%ID at 30 minutes to 28 ± 1.6%ID at 240 minutes) further substantiates efficient renal clearance and reinforces the kidneys as the principal elimination pathway [56].

**Table 4:** Biological evaluation study of [^131^I]Cefa in normal mice, expressed as %ID/organ post injection, (X ± S.D., *n* = 7)

Organs & body fluids	[^131^I]Cefa in normal mice at different time intervals post injection (min)			
	30 min	60 min	120 min	240 min
Bone	2.5 ± 0.03	2.2 ± 0.05	1.5 ± 0.2	0.8 ± 0.3
Heart	2.4 ± 0.02	1.7 ± 0.01	1.2 ± 0.01	0.6 ± 0.01
Lungs	1.4 ± 0.05	1.2 ± 0.04	0.8 ± 0.03	0.6 ± 0.05
Liver	2.7 ± 0.4	3.4 ± 0.3	2.0 ± 0.2	1.8 ± 0.2
Spleen	0.4 ± 0.01	0.3 ± 0.01	0.2 ± 0.01	0.1 ± 0.01
Kidneys	7.5 ± 0.9	9.8 ± 1.3	4.7 ± 1.1	4.1 ± 1.0
Stomach	12 ± 1.0	15 ± 1.5	12 ± 1.4	6.5 ± 1.0
Intestine	7.1 ± 1.3	8.5 ± 1.4	16 ± 1.7	10 ± 1.2
Thyroid	1.5 ± 0.1	1.4 ± 0.1	1.2 ± 0.07	0.8 ± 0.05
Urine	8.0 ± 1.1	17 ± 1.2	22 ± 1.4	28 ± 1.6
*Blood*	*28 ± 1.4*	*25 ± 1.5*	*10.5 ± 1.0*	*3.8 ± 1.2*
*Muscle (N)*	*12± 1.2*	*10.8 ± 1.1*	*8.5± 0.8*	*4.5 ± 0.5*

Low hepatic uptake at 60 minutes, 3.4 ± 0.3%ID, and decreased with time passing, reaching 1.8 ± 0.2%ID at 240 minutes, while intestinal uptake manifested a delayed peak, escalating from 7.1 ± 1.3%ID at 30 minutes to a maximum of 16 ± 1.7%ID at 120 minutes. This temporal profile suggests a secondary accumulation phase, potentially attributable to hepatobiliary excretion and enterohepatic recirculation. The sustained intestinal retention at later intervals may also stem from partial reabsorption or binding of [^131^I]Cefa to mucosal surfaces, consonant with cefaclor’s documented gastrointestinal interactions and partial biliary elimination. The stomach showed moderate retention, particularly at 60 minutes (15 ± 1.5%ID), which may reflect altered gastric mucosal dynamics and binding with [^131^I]Cefa. In addition, the gastric localization of [^131^I]Cefa can be rationalized by previously reported findings that cefaclor delays gastric emptying through capsaicin-sensitive afferent pathways mediated by CCKA receptors [57, 58]. This mechanism is attributed to the stimulation of cholecystokinin (CCK) secretion from intestinal endocrine Icells, which subsequently inhibits gastric emptying and prolongs retention of the compound within the stomach. Furthermore, cefaclor possesses a peptidomimetic structure analogous to peptones, which are well known to induce CCK release. Such structural similarity provides a plausible explanation for the observed gastric accumulation of the radiolabeled derivative, aligning with established physiological pathways and supporting the interpretation of tracer retention in this organ [59–61].

Muscle tissues exhibited minimal retention across the study duration, with a consistent decline from 12 ± 1.2%ID at 30 minutes to 4.5 ± 0.5%ID at 240 minutes. This modest and diminishing uptake denotes negligible affinity of [^131^I]Cefa for normal muscle tissue, underscoring its restricted extravascular distribution in non-targeted peripheral compartments. The lack of substantial muscle accumulation corroborates the compound’s hydrophilic characteristics and expeditious clearance from non-metabolizing tissues.

Thyroid uptake remained consistently low across all evaluated time points, measuring 1.5 ± 0.1%ID, 1.4 ± 0.1%ID, 1.2 ± 0.07%ID, and 0.8 ± 0.1%ID at 30, 60, 120, and 240 minutes, respectively. This limited accumulation in thyroid tissue serves as a critical indicator of the in vivo stability of the radiolabeled compound [^131^I]Cefa, strongly suggesting that the radioisotope remains securely bound to the cefaclor structure without undergoing deiodination. The observed low thyroid uptake for [^131^I]Cefa attests to the chemical integrity of the radiolabeled entity and mitigates concerns regarding the release of free iodine, a crucial consideration for the safety profile of radiopharmaceuticals [61]. This observation aligns with the in vitro metabolic stability evaluation, wherein [^131^I]Cefa maintained 89.5 ± 0.5% integrity at 4 hours, thereby reinforcing its resistance to deiodination. Such stability is essential for predicting the compound’s interaction with metabolic pathways in vivo and for ensuring reliable pharmacokinetic performance.

In comparison with [^131^I]Cefa to the iodotyramine derivative (compound 27), the metabolic stability of [^131^I]Cefa is particularly noteworthy. Specifically, thyroid uptake of [^131^I]Cefa reached 1.4 ± 0.1%ID at 60 minutes and decreased to 0.8 ± 0.1%ID per organ at 240 minutes. Conversely, compound 27 exhibited significantly higher thyroid accumulation, with values of 3.2 ± 0.98%ID per organ at 1 hour increasing to 14 ± 2%ID per organ at 3 hours. This increasing pattern indicates metabolic degradation of compound 27, resulting in the release of free iodine and subsequentlyleading to undesirable thyroid accumulation. Collectively, these observations confirm that [^131^I]Cefa demonstrates a metabolic stability both in vitro and in vivo. Collectively, these findings confirm the pharmacokinetic profile of [^131^I]Cefa, marked by rapid blood clearance, predominant renal and urinary excretion, moderate hepatic metabolism, and minimal retention in thyroid or non-target tissues. One-way ANOVA (*p* < 0.05) further demonstrated significant time-dependent differences in organ uptake, reinforcing its efficient clearance and limited off-target accumulation.

**Fig. 10:**
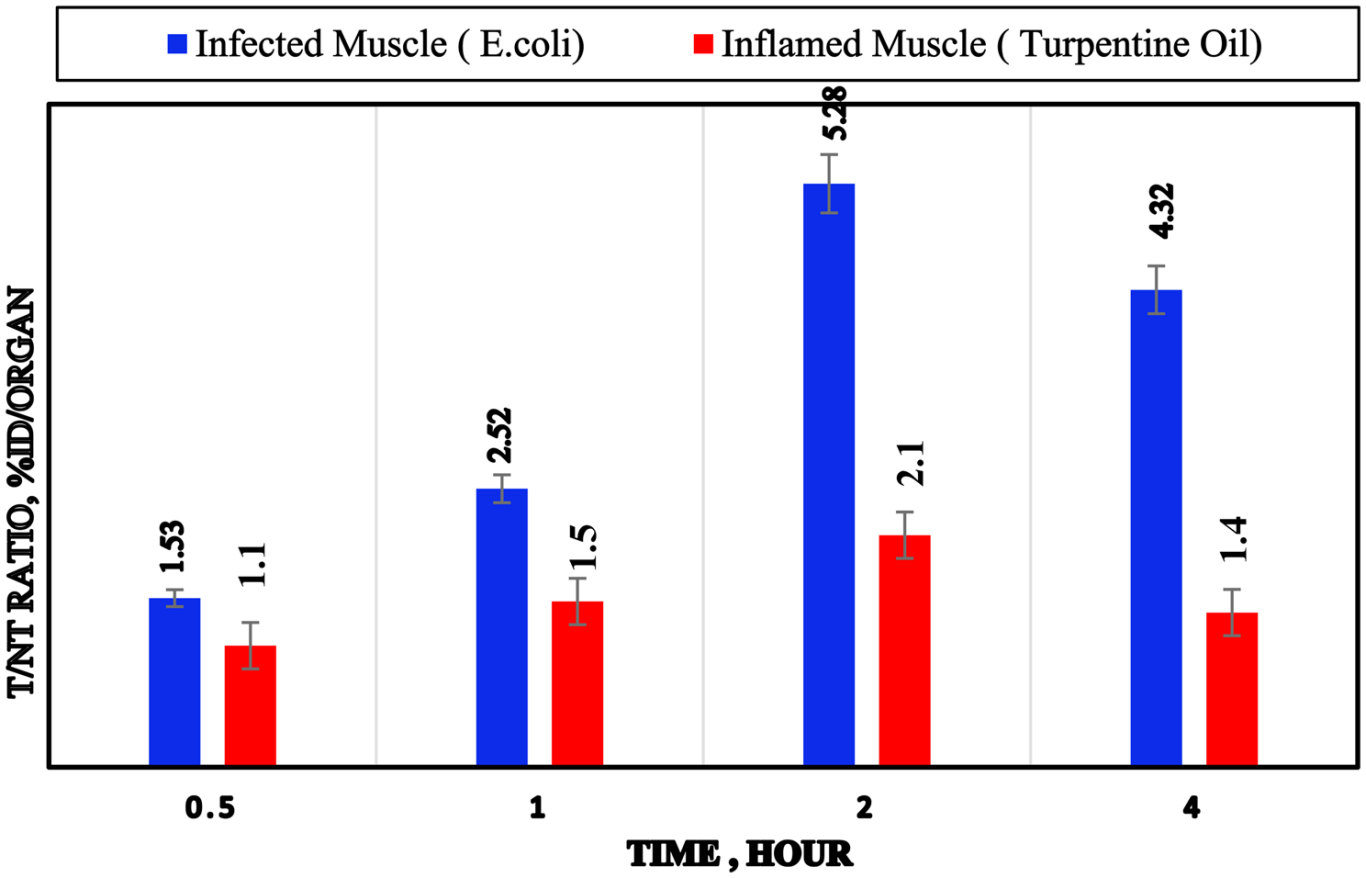
Comparison of [^131^I]Cefa tracer uptake of the target/blood ratio in infected, and inflamed models

**Fig. 11:**
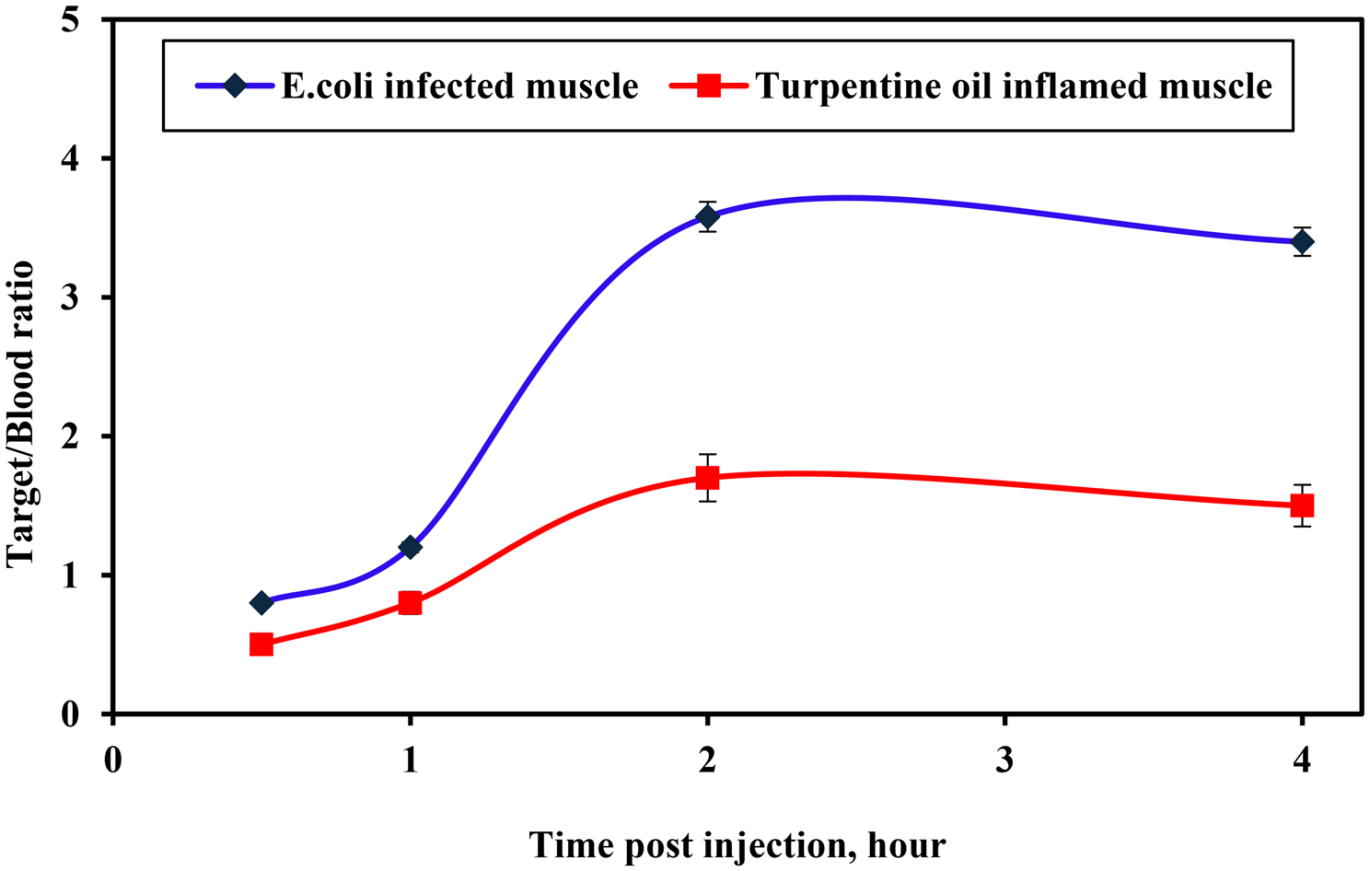
The T/NT ratios of [^131^I]Cefa tracer uptake in infected, and inflamed models over time

The biodistribution profile of [^131^I]Cefa in *E. coli*-infected mice reveals significant deviations in organ uptake compared to normal physiological conditions, indicating the compound’s sensitivity to infection-related pathophysiological changes, as detailed in Table 5. Notable differences were observed in uptake between infected (target) and uninfected (non-target) muscle tissues. The infected muscle exhibited substantially increased accumulation, reaching a peak of 28 ± 1.5%ID at 120 minutes post-injection, in contrast to 5.3 ± 0.6%ID in the non-target muscle at the same time. Application of the Student’s unpaired t-test (*p* < 0.05) confirmed statistically significant differences in uptake between infected and normal muscle across various time intervals. This selective retention is corroborated by the target-to-non-target (T/NT) ratio, which increased from 1.53 at 30 minutes to a maximum of 5.28 at 120 minutes, as shown in Fig. 10, indicating. pronounced preferential localization within the infected tissue. Similarly, the target-to-blood (T/Bl) ratio rose from 0.8 to 3.58 over the corresponding timeframe, denoting enhanced target-to-background contrast, as illustrated in Fig. 11.

**Table 5:** Biological evaluation study of [^131^I]Cefa in infected mice with *E. coli*, expressed as %ID/organ post injection (X ± S.D., *n* = 7)

Organs & body fluids	[^131^I]Cefa in infected mice with E. coli at different time intervals post-injection (min)			
	30 min	60 min	120 min	240 min
Bone	2.7 ± 0.3	2.4 ± 0.1	2.2 ± 0.3	0.7 ± 0.3
Heart	2.8 ± 0.05	1.8 ± 0.05	1.5 ± 0.03	0.7 ± 0.03
Lung	1.8 ± 0.1	1.4 ± 0.1	1.2 ± 0.2	0.8 ± 0.1
Liver	3.5 ± 0.2	7.8 ± 0.6	4.5 ± 0.3	2.0 ± 0.1
Spleen	0.4 ± 0.01	0.3 ± 0.01	0.2 ± 0.01	0.1 ± 0.01
Kidneys	8.5 ± 1.1	16 ± 1.3	14 ± 1.2	10.6 ± 0.9
Stomach	13 ± 1.4	18 ± 1.5	14.5 ± 1.2	7.6 ± 1.1
Intestine	8.8 ± 1.2	10.6 ± 1.3	18 ± 1.5	13.5 ± 1.2
Thyroid	1.4 ± 0.04	1.3 ± 0.03	1.0 ± 0.02	0.7 ± 0.01
Urine	10 ± 1.3	18 ± 1.5	25 ± 1.2	32 ± 1.6
*Blood*	*25 ± 1.1*	*20± 1.2*	*7.8± 1.0*	*4.7± 0.7*
*Inf. Muscle (T)*	*20 ±1.1*	*24 ±1.2*	*28 ± 1.5*	*16 ± 1.1*
*Muscle (NT)*	*13 ± 0.6*	*9.5 ± 0.5*	*5.3 ± 0.6*	*3.7 ± 0.4*
*T/NT Ratio*	*1.53*	*2.52*	*5.28*	*4.32*
*T/Bl Ratio*	*0.8*	*1.2*	*3.58*	*3.4*

T: Target (Infected Muscle); NT: Non-Target (Normal Muscle); Bl: Blood

The localization of [^131^I]Cefa in the infected thigh muscle can be explained by its pharmacological mechanism and the pathophysiological modifications induced by infection. As a second-generation cephalosporin, cefaclor exhibits bactericidal efficacy by binding to penicillin-binding proteins (PBPs) and inhibiting peptidoglycan cross-linking, thereby causing bacterial cell wall disruption and lysis. In addition to this inherent antimicrobial property, the observed tracer accumulation in infected tissue is likely influenced by infection-mediated factors, including increased vascular permeability, localized inflammatory exudation, altered perfusion dynamics, and prolonged retention within the inflamed environment [19]. These mechanisms facilitate enhanced delivery and sequestration of the radiolabeled compound at infection sites. The clear difference in uptake between infected and normal muscle tissues indicates that [^131^I]Cefa has the ability to distinguish infection sites, suggesting its potential use as a radiodiagnostic agent for infection imaging.

The biological evaluation data profile of [^131^I]Cefa in mice subjected to sterile inflammation induced by turpentine oil reveals a pharmacokinetic profile influenced by localized sterile inflammation, as presented in Table 6. Notably, there is a differential uptake between inflamed (target) and normal (non-target) muscle tissues. The inflamed muscle exhibited higher accumulation, peaking at 16 ± 1.5%ID at 120 minutes, compared to 7.5 ± 0.6%ID in the non-target muscle. According to the Student’s unpaired test (*p* < 0.05), the difference in uptake between the inflamed and normal muscle was significant across various time intervals. This selective retention is quantitatively supported by the T/NT ratio, which increased from 1.1 at 30 minutes to 2.1 at 120 minutes, indicating preferential localization in inflamed tissue. The T/Bl ratio also rose from 0.5 to 1.7 over the same interval, enhancing target-to-background contrast. These findings suggest that [^131^I]Cefa possesses inflammation-targeting properties, likely mediated by increased vascular permeability, enhanced retention, and altered tissue diffusion into inflamed tissues. In conclusion, [^131^I]Cefa demonstrates favorable biodistribution in sterile inflammation models, with rapid blood clearance, elevated renal processing, minimal thyroid uptake, and selective accumulation in inflamed muscle. These properties support its potential utility as a radiopharmaceutical agent for imaging sterile inflammatory lesions and assessing tissue-specific drug distribution.

**Table 6:** Biological evaluation study of [^131^I]Cefa in inflamed mice with sterile turpentine oil, expressed as %ID/organ post injection (X ± S.D., *n* = 7)

Organs & body fluids	[^131^I]Cefa in inflamed mice with sterile turpentine oil at different time intervals post-injection (min)			
	30 min	60 min	120 min	240 min
Bone	2.6 ± 0.4	2.3 ± 0.3	2.2 ± 0.3	1.8 ± 0.2
Heart	2.6 ± 0.5	1.6 ± 0.4	1.4 ± 0.3	0.9 ± 0.08
Lung	1.2 ± 0.01	1.1 ± 0.01	1.0 ± 0.02	0.9 ± 0.01
Liver	3.2 ± 0.3	7.2 ± 0.5	5.0 ± 0.4	2.2 ± 0.2
Spleen	0.5 ± 0.02	0.4 ± 0.01	0.3 ± 0.02	0.2 ± 0.01
Kidneys	9.5 ± 1.1	15.8 ± 1.4	12.9 ± 1.3	10.6 ± 1.2
Stomach	14.7 ± 1.4	17.2 ± 1.5	11.2 ± 1.3	8.0 ± 1.2
Intestine	10 ± 1.0	12 ± 1.2	16 ± 1.3	11 ± 1.1
Thyroid	1.3 ± 0.08	1.2 ± 0.07	0.9 ± 0.07	0.6 ± 0.05
Urine	6.5 ± 1.1	9.0 ± 1.1	14.5 ± 1.2	26 ± 1.6
*Blood*	*22 ± 1.4*	*16 ± 1.3*	*9.0± 1.2*	*4.5±1.1*
*Infl. Muscle (T)*	*11± 0.9*	*13±1.3*	*16± 1.5*	*7.0±1.1*
*Muscle (NT)*	*10 ± 0.9*	*8.2 ± 0.8*	*7.5 ± 0.6*	*5.0 ± 0.5*
*T/NT Ratio*	*1.1*	*1.5*	*2.1*	*1.4*
*T/Bl Ratio*	*0.5*	*0.8*	*1.7*	*1.5*

T: Target (Inflamed Muscle); NT: Non-Target (Normal Muscle); Bl: Blood

The evaluation of [^131^I]Cefa indicates improved biodistribution and higher target-to-non-target (T/NT) ratios compared with several previously reported tracers. For example, while ^99 m^ Tc-ciprofloxacin typically shows T/NT ratios between 3.0 and 3.5 in similar animal models, [^131^I]Cefa reached 5.28 at 120 minutes post-injection. Molecular docking simulations suggested binding affinity to bacterial DNA gyrase B, which may contribute to the observed selective uptake in infected tissues. At two hours post-injection, uptake in infected muscle (28 ± 1.5%ID/organ) was approximately 1.5-fold higher than in sterile inflamed tissue, supporting its ability to distinguish bacterial infection from aseptic inflammation. In vitro stability studies further showed that [^131^I]Cefa remained 89.5 ± 0.5% intact at 4 hours, with low thyroid uptake, indicating resistance to deiodination. Compared with tracers such as ^131^I-ornidazole (T/NT = 2.9 at 5 h) [8], typically ^99 m^ Tc-vancomycin (T/NT = 5 at 1 h) [14], typically ^99 m^ Tc-cefoperazone (T/NT = 4.5 at 45 min) [15], typically ^99 m^ Tc-kanamycin (T/NT = 1.75 at 2 h) [16], typically ^99 m^ Tc-alafosfalin (T/NT = 4.3 at 4 h) [17], and the commercial exactly ^99 m^ Tc-ciprofloxacin (T/NT = 3.18 at 1 h) [18], [^131^I]Cefa demonstrates relatively higher stability and selectivity for infectious imaging.

Overall, these findings suggest that [^131^I]Cefa provides advantages in infection imaging, combining acceptable physicochemical stability with improved biological performance. Although the T/NT ratio was elevated in infected mice compared to inflamed mice, the difference did not achieve statistical significance (*p* < 0.05), despite the infected group exhibiting an upward trend. In conclusion, [^131^I]Cefa serves as an effective tracer for imaging or targeting infections, as radiolabeled antibiotics inherently localize at infection sites by exploiting pathophysiological changes such as increased blood flow, enhanced vascular permeability, and the subsequent extravasation of circulating tracers into infected and inflamed tissues. Infected sites frequently exhibit compromised vascular integrity and inflammatory hyperemia, facilitating preferential tracer delivery and retention relative to adjacent normal tissue. This mechanism underpins numerous radiolabeled drug strategies for detecting infection and inflammation in nuclear medicine.

## Conclusion

The radioiodination of cefaclor using iodogen on glass frits as the oxidizing system achieved a maximum radiolabeling efficiency of 90 ± 0.56%. The radiolabeled compound was subsequently purified through HPLC before biological evaluation in murine models of inflammation. In vivo biodistribution studies demonstrated the diagnostic potential of [^131^I]Cefa as a radiotracer for both septic and aseptic infections, with significant tracer accumulation in infected and inflamed tissues, measuring 28 ± 1.5% and 16 ± 1.5%ID/organ at 2 hours post-injection, respectively. These findings were further substantiated by elevated T/NT ratios of 5.28 and 2.1 in septic and aseptic models, respectively, indicating strong site-specific localization at 120 minutes post-injection. Complementary molecular docking analyses confirmed the affinity of [^131^I]Cefa for *bacterial DNA gyrase B*, supporting its mechanism of action and reinforcing its suitability as a targeted imaging agent for infectious and inflammatory pathologies.

## Data Availability

All data generated or analyzed during this study are included in the manuscript.

## Abbreviation

*E.**coli*: Escherichia coli
CAT: Chloramine-T [N-chloro-p-toluene sulfonamide salt]
NBS: N-N-bromosuccinamide
Iodogen: (1,3,4,6-tetrachloro-3,6-diphenyl glycoluril)
TLC: Thin-layer chromatography (TLC) aluminum sheets
HPLC: High Performance Liquid Chromatography
CFU: Colony-Forming Units
DMSO: Dimethyl Sulfoxide
DMF: Dimethyl Formamide
MBq: Mega Becquerel
MOE: Molecular Operating Environment
E: Energy
PDB: Protein Data Bank
T: Target (Infected/Inflamed) Muscle
NT: Non-Target (Normal) Muscle
Bl: Blood
